# *OsFPFL4* is Involved in the Root and Flower Development by Affecting Auxin Levels and ROS Accumulation in Rice (*Oryza sativa*)

**DOI:** 10.1186/s12284-019-0364-0

**Published:** 2020-01-07

**Authors:** Yaomin Guo, Qi Wu, Zizhao Xie, Bo Yu, Rongfeng Zeng, Qian Min, Junli Huang

**Affiliations:** Key Laboratory of Biorheological Science and Technology (Chongqing University), Ministry of Education, Bioengineering College, Chongqing, 400030 China

**Keywords:** Auxin, Flower, *OsFPFL4*, Root, ROS

## Abstract

**Background:**

*FPF1* (*flowering-promoting factor 1*) is one of the important family involved in the genetic control of flowering time in plant. Until now, limited knowledge concerning *FPF1* family in rice has been understood.

**Results:**

As a homologue of *AtFPF1*, *FPF1-like protein 4* of rice (*OsFPFL4*) is expressed in various tissues of plants. The functions of *OsFPFL4* in rice were investigated by the reverse genetics approaches. Plants overexpressing *OsFPFL4* have shorter primary root, more lateral roots and adventitious roots than wild type; however, RNA interference (RNAi) of *OsFPFL4* significantly inhibits the growth of root system, and also delays the flowering time in rice. Interestingly, increased or repressed expression of *OsFPFL4* leads to shrunken anthers and abnormal pollen grains. It is well recognized that auxin plays important roles in plant root and flower development, and the root elongation is also regulated by reactive oxygen species (ROS) homeostasis. Here, our results show that rice plants overexpressing *OsFPFL4* accumulate more auxin in the shoot and root, whereas RNAi lines have less auxin than wild type. As expected, the transcript levels of genes responsible for auxin biosynthesis and polar transport are altered in these *OsFPFL4* transgenic plants. As to ROS, slightly higher ROS levels were detected in overexpression root and inflorescence than the counterparts of wild type; however, the ROS levels were significantly increased in the RNAi lines, due to increased expression of ROS-producers and reduced expression of ROS-scavengers.

**Conclusion:**

Our results reveal that *OsFPFL4* is involved in modulating the root and flower development by affecting auxin and ROS homeostasis in rice plants. *OsFPFL4* controls auxin accumulation via affecting auxin biosynthesis and transport, and also modulates ROS homeostasis by balancing ROS producing and scavenging. Thus, auxin-mediated ROS production might play a role in regulating redox status, which controls plant root and flower development.

## Background

Promoted root system architecture is crucial for crop growth performance and productivity (Van Norman et al. 2013). Most monocotyledonous plants have fibrous root systems composed of the adventitious root and lateral root (Jiang et al. 2017; Huang et al. 2018). Primary root development starts during embryogenesis. Lateral roots initiating from the primary root play critical roles in plant root development, since they determine the architecture of the root system and maximize the potential of the root system for water and nutrient uptake (Hochholdinger et al. 2004; Parizot et al. 2008; Coudert et al. 2010). As a post-embryonic mode of organogenesis in plant, development of lateral roots is a typical example of de novo organogenesis, according to a regular pattern (De Smet et al. 2006). Plant hormone auxin directs many developmental responses, including the elaboration of branching patterns in the root (Guseman et al. 2015). Endogenous auxin biosynthesis, polar transport, and degradation/conjugation can change its accumulation and auxin-dependent signaling (Petricka et al. 2012; Lavenus et al. 2013), and mutations involved in these processes significantly affect the lateral root development (Fukaki and Tasaka 2009; Peret et al. 2009). For instance, gain-of-function mutants such as *yucca1*-*D*, *sur1*, and *sur2* with increased auxin levels produce more lateral roots (Zhao et al. 2001; Peret et al. 2009). By contrast, mutants with altered auxin transport, such as Arabidopsis *aux1*, *lax3*, *pin1*, *pin 3/7*, *pin 4/7* and rice *osaux1* have less lateral roots (Benkova et al. 2003; Peret et al. 2009; Zhao et al. 2015).

Although reactive oxygen species (ROS) such as superoxide anions (O_2_^−^) and hydrogen peroxide (H_2_O_2_) are generally considered to be toxic to cells, a series of evidence proved that ROS at appropriate levels function as ubiquitous signaling molecules to regulate plant development and stress adaptation (Xia et al. 2009; Ivanchenko et al. 2013). Specifically, ROS produced in the apoplast by NADPH oxidase, encoded by *RESPIRATORY BURST OXIDASE HOMOLOGS* (*RBOH*s), play diverse roles in the regulation of seed germination (Mueller et al. 2009), development of pollen tubes (Potocky et al. 2007), and bud outgrowth (Chen et al. 2016). ROS also act a part in the root development. Recently, an increasing number of evidence showed that ROS function in the regulation of root meristem activity (Yang et al. 2014), root cell differentiation (Tsukagoshi et al. 2010), root elongation (Tsukagoshi et al. 2010; Chen et al. 2016; Liu et al. 2016), lateral root emergence (Orman-Ligeza et al. 2016), and root hair formation (Sundaravelpandian et al. 2013). In another report, *PvRbohB* silencing reduces the lateral root density in transgenic *Phaseolus vulgaris* roots (Montiel et al. 2013), implying that hormone-controlled developmental events are mediated by *RBOH*s. In addition, the pool of apoplast ROS can be modulated by the activity of class III peroxidases (PERs), which enable wall loosening by generating ROS (Passardi et al. 2004). It has been described that auxin-mediated developmental processes are closely associated with ROS production (Xia et al. 2015). Auxin-induced ROS are directly involved in cell-wall loosening and have a crucial role in cell elongation (Schopfer 2001), and overexpression of the cell-wall-localized ascorbate oxidase gene results in increased oxidation of the apoplast and mimics auxin-mediated effects on plant growth (Pignocchi et al. 2003). Moreover, the increased cellular oxidation state associated with auxin maxima is thought to arrest the cell cycle in the quiescent center, which is important for the maintenance of the root meristem (Jiang and Zhang 2003; Heyman et al. 2013).

Flowering and floral development are very important traits for cultivars in agriculture since they impact crop yield. *FLOWERING PROMOTING FACTOR 1* from mustard (*MuFPF1*) was originally understood based on its role in flowering, and overexpression of *MuFPF1* promoted flowering time in Arabidopsis (Melzer et al. 1990; Kania et al. 1997). Up to now, homologous genes of *MuFPF1* have been characterized in Arabidopsis (Melzer et al. 1999), rice (*Oryza sativa*) (Ge et al. 2004), tobacco (*Nicotiana tabacum*) (Smykal et al. 2004), and cotton (*Gossypium* L.) (Wang et al. 2014), which have been shown to confer promotion of flowering time. Similar to *MuFPF1*, constitutive expression of *AtFPF1* can lead to early flowering in Arabidopsis (Melzer et al. 1999). Additionally, introduction of *AtFPF1* into rice also conferred early flowering, suggesting that it is involved in the genetic control of flowering time in both dicots and monocots (Xu et al. 2005). It is notable that rice plants overexpressing *AtFPF1* also had more adventitious roots and shorter primary and adventitious roots than wild type (Xu et al. 2005). Overexpression of cotton *GhFPF1* in Arabidopsis promoted flowering time and shade-avoidance responses (Wang et al. 2014). In rice, a homologue of *AtFPF1*, *OsRAA1* (*FPF1-like 1*)*,* has been characterized. Different from *AtFPF1*, *OsRAA1* did not obviously modulate flowering time in rice, but was involved in auxin-mediated flower and root development (Ge et al. 2004). In summarize, *FPF1* gene family takes part in several aspects of plant development. In spite of these progresses, limited knowledge concerning the underlying mechanism of *FPF1* family in plant is understood.

In this report, we investigated the developmental roles of another novel *FPF1* homologue in rice, *OsFPFL4* (*FPF1-like 4*), by reverse genetics approaches. We propose that auxin-induced ROS production might play crucial roles in the developmental processes performed by *OsFPFL4*.

## Results

### *OsFPFL4* is a Homologue of *FPF1*

**Fig. 1:**
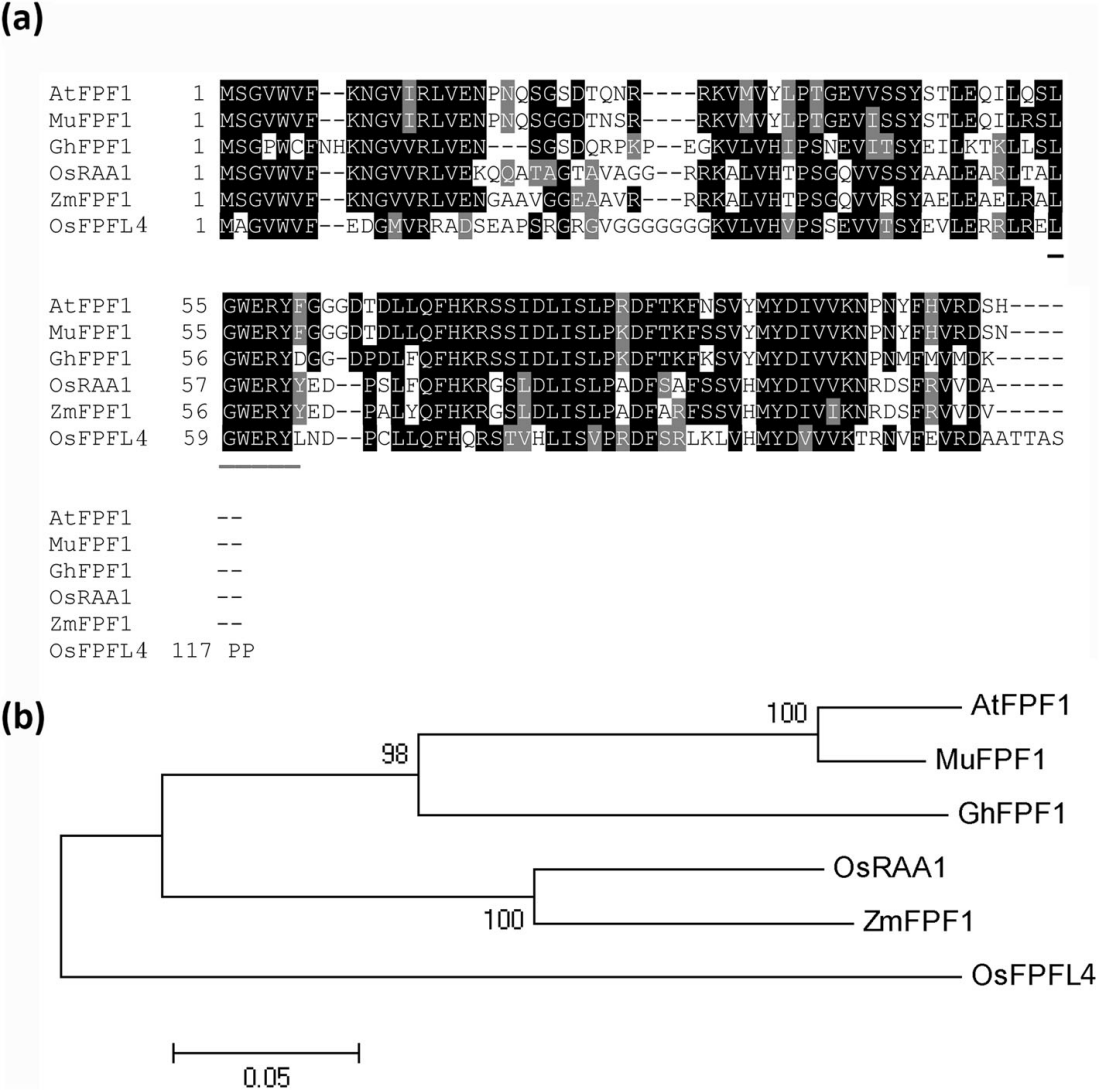
Comparative analysis of OsFPFL4 and other FPF1 homologues. **a** Multiple alignment of FPF1 protein sequences in several species. AtFPF1 (Y11988) from *Arabidopsis thaliana*; MuFPF1 (Y11987) from mustard (*Sinapis alba*), NtFPF1 (AY496934) from (*Nicotiana tabacum*), ZmFPF1 (ACG44143) from maize (*Zea mays*), and OsRAA1 (AY659938) and OsFPFL4 (AK120187) from rice (*Oryza sativa*). **b** Phylogenetic tree of the FPF1 proteins in the above plants as determined by the MEGA 5.0 software package

There are five *FPF1*-*like* genes in rice (denoted as *FPF1-like 1–5*) (Additional file 3: Figure S1). Here, *OsFPFL4* (*FPF1-like 4*), a novel *FPF1* homologue in rice, was studied. Multiple alignment of amino acid sequences of a series of *FPF1* homologues, *MuFPF1* from white mustard (*Sinapis alba*), *AtFPF1* from *Arabidopsis thaliana*, *OsRAA1* from rice (*Oryza sativa*), *NtFPF1* from tobacco (*Nicotiana tabacum*), *ZmFPF1* from maize (*Zea mays*) and *GhFPF1* from cotton (*G. hirsutum* L.), revealed that OsFPFL4 shared high similarity to other FPF1 proteins (Fig. 1a). The result also indicated that there was at least one conserved domain, −LGWERY-, present in this small protein family (Fig. 1a). Phylogenetic analysis of FPF1 homologues indicated OsFPFL4 was located in a branch close to OsRAA1, but far from AtFPF1 (Fig. 1b), implying that *OsFPFL4* might have functions similar to that of *OsRAA1* in plant growth and development.

### Transcription Patterns of *OsFPFL4*

**Fig. 2:**
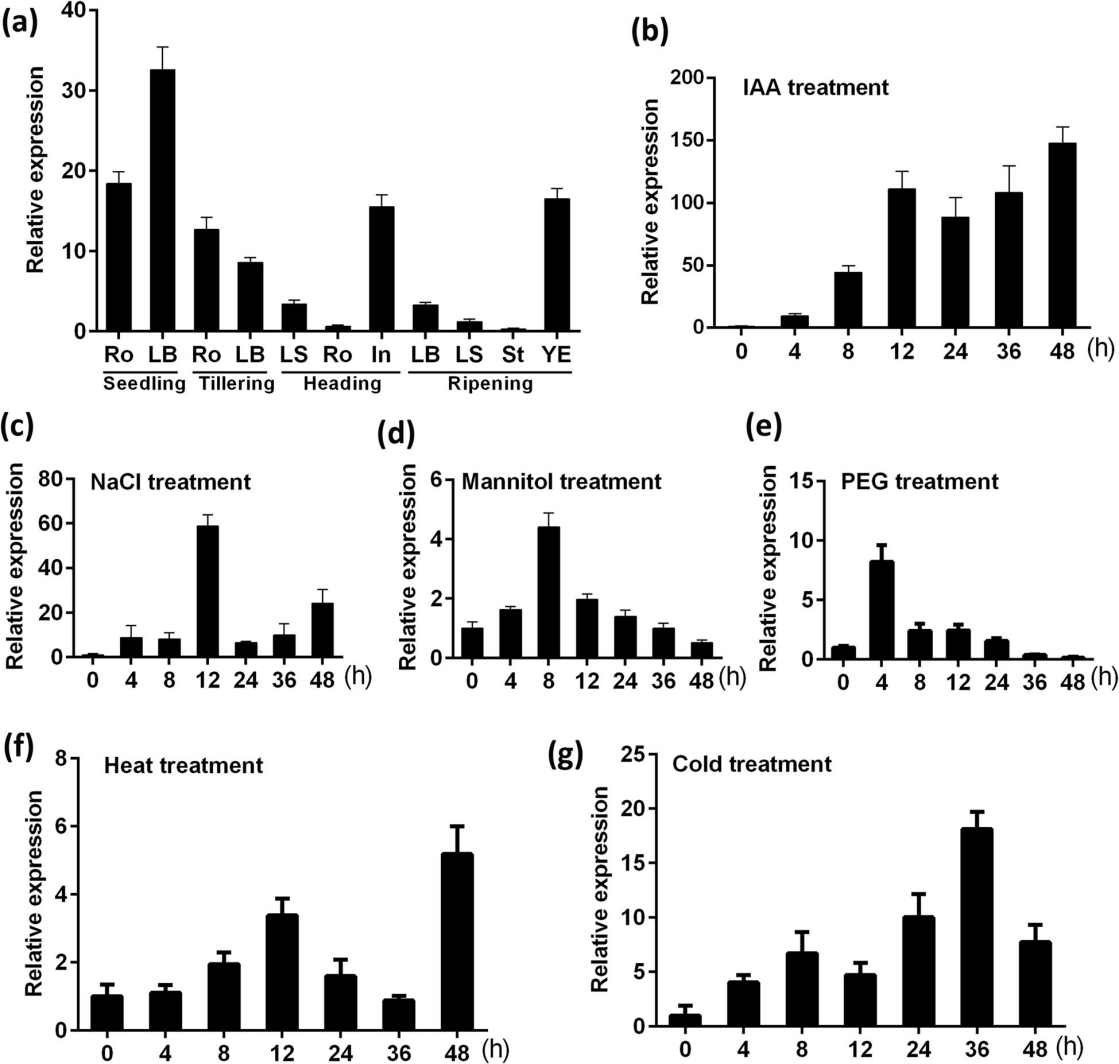
Expression profiles of *OsFPFL4* determined by quantitative PCR analysis. **a** Temporal and spatial expression patterns of *OsFPFL4.* Time-courses of *OsFPFL4* expression in response to hormone of 20 μM IAA (**b**) and abiotic stresses such as 200 mM NaCl (**c**), 100 mM mannitol (**d**), 20% PEG6000 (**e**), heat (42 °C) (**f**) or cold (4 °C) (**g**), respectively, in 10-day-old seedlings. Seedlings grown in standard 1/2 MS liquid medium under normal conditions were used as the control. Leaf samples were used for quantitative real-time PCR analysis. Error bars indicate standard error (*n* = 3). Three replica experiments were performed. Seedling, grown for 1 week; Tillering, grown for 1 month; Heading, grown for 2 months; Ripening, grown for 3 months. Ro, root; LB, leaf blade; LS, leaf sheath; In, inflorescence; St, stem; YE, young embryo

To gain more insight into the genetic control of *OsFPFL4* in plant development in rice, we examined its spatio*-* and temporal*-*transcription patterns. *OsFPFL4* mRNA preferentially accumulated in the leaf blade and root at the seedling as well as tillering stages; however, at the heading stage, transcripts of *OsFPFL4* peaked in the inflorescence, and only small quantities of transcripts accumulated in the leaf blade and leaf sheath (Fig. 2a). Scant transcripts of *OsFPFL4* were detected in other organs except in young embryo at the ripening stage (Fig. 2a). These findings imply that *OsFPFL4* might be involved in the growth and development of different organs of rice plants.

The responses of plant to environmental cues play an important role in adaption to the abiotic stress, and hormones are involved in the regulation of the developmental processes in plant. We therefore analyzed the response of *OsFPFL4* in the seedlings treated by hormones or abiotic restraints. Remarkable response of *OsFPFL4* to hormone IAA was observed, and the mRNA levels of *OsFPFL4* were greatly induced by IAA (Fig. 2b). A significant increase in the transcription of *OsFPFL4* was observed when rice seedlings were treated by NaCl, mannitol or PEG (Fig. 2c-e), implying its roles in osmotic tolerance. Additionally, *OsFPFL4* expression was also significantly induced in response to temperature stress (Fig. 2f and g). Overall, the expression patterns of *OsFPFL4* indicate that it might be involved in plant growth and development and is therefore likely to be crucial for improving plant tolerance to external restraints.

### *OsFPFL4* is Involved in the Development of Root System and Plant Growth

**Fig. 3:**
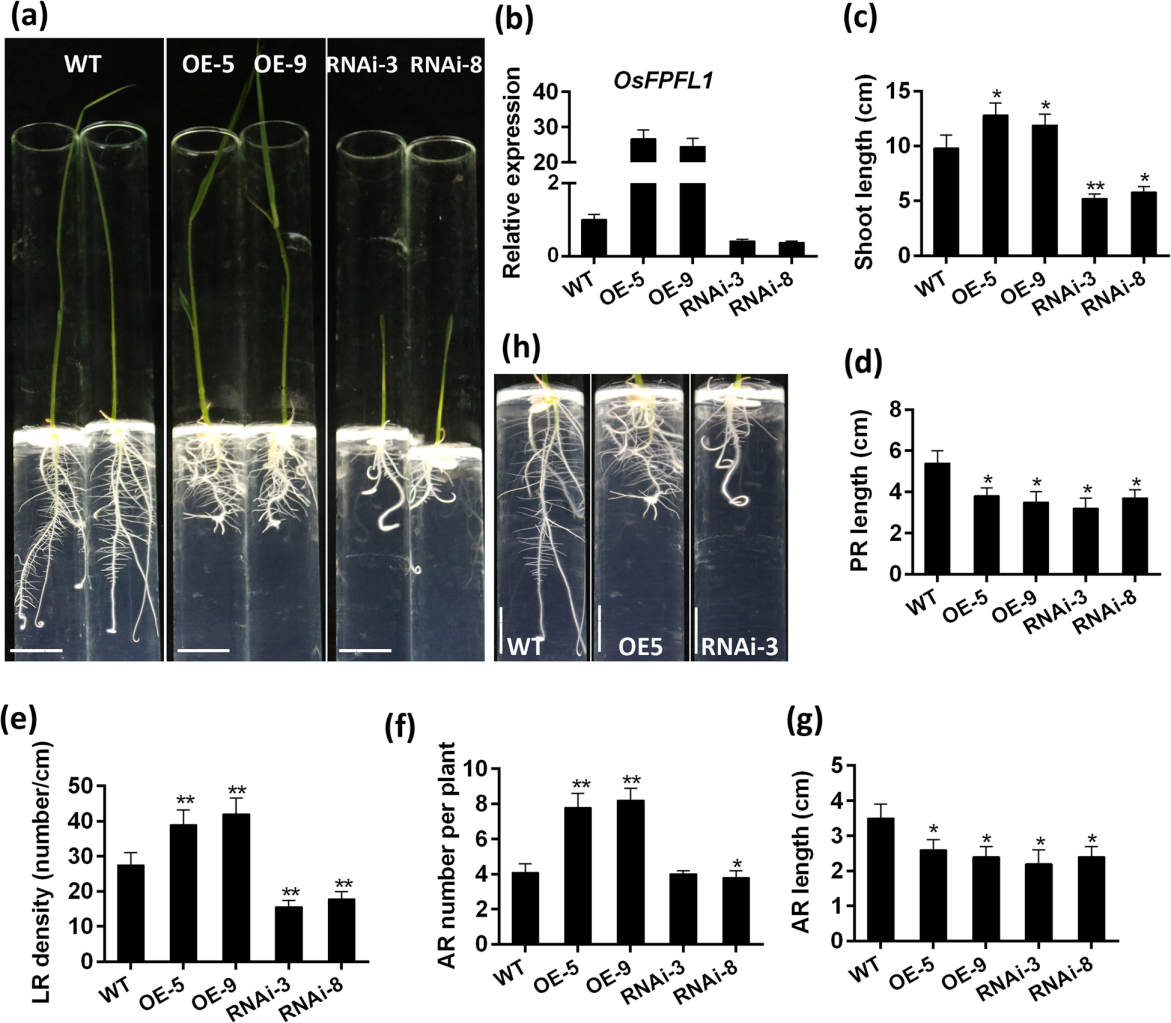
Phenotypes of *OsFPFL4* transgenic lines are correlated with *OsFPFL4* transcript levels. **a** Seven-day-old wild type (WT) and *OsFPFL4* transgenic seedlings in 1/2 MS agar in tubes. Scale bars, 2 cm. **b** Analysis of *OsFPFL4* transcript levels in 7-day-old seedlings from independent transgenic lines and WT by quantitative real-time PCR. Error bars indicate standard error (*n* = 3). Three replica experiments were performed. **c**-**g** Shoot length, PR length, LR density, AR number per plant and average AR length in 7-day-old WT and transgenic seedlings. Uniformly germinated were grown in 1/2 MS agar medium in tubes for 7 days in a growth chamber. **h** Root systems of 7-day-old WT and transgenic plants. Scale bars, 1 cm. WT, wild type. OE-5 and OE-9, *OsFPFL4* overexpression transgenic lines. RNAi-3 and RNAi-8, *OsFPFL4*-RNAi transgenic lines. Data are means ± SE (*n* = 15–30). The significant difference between *OsFPFL4* transgenic lines and wild type was determined by Student’s *t*-test. ^*^p < 0.05, ^**^p < 0.01

**Fig. 4:**
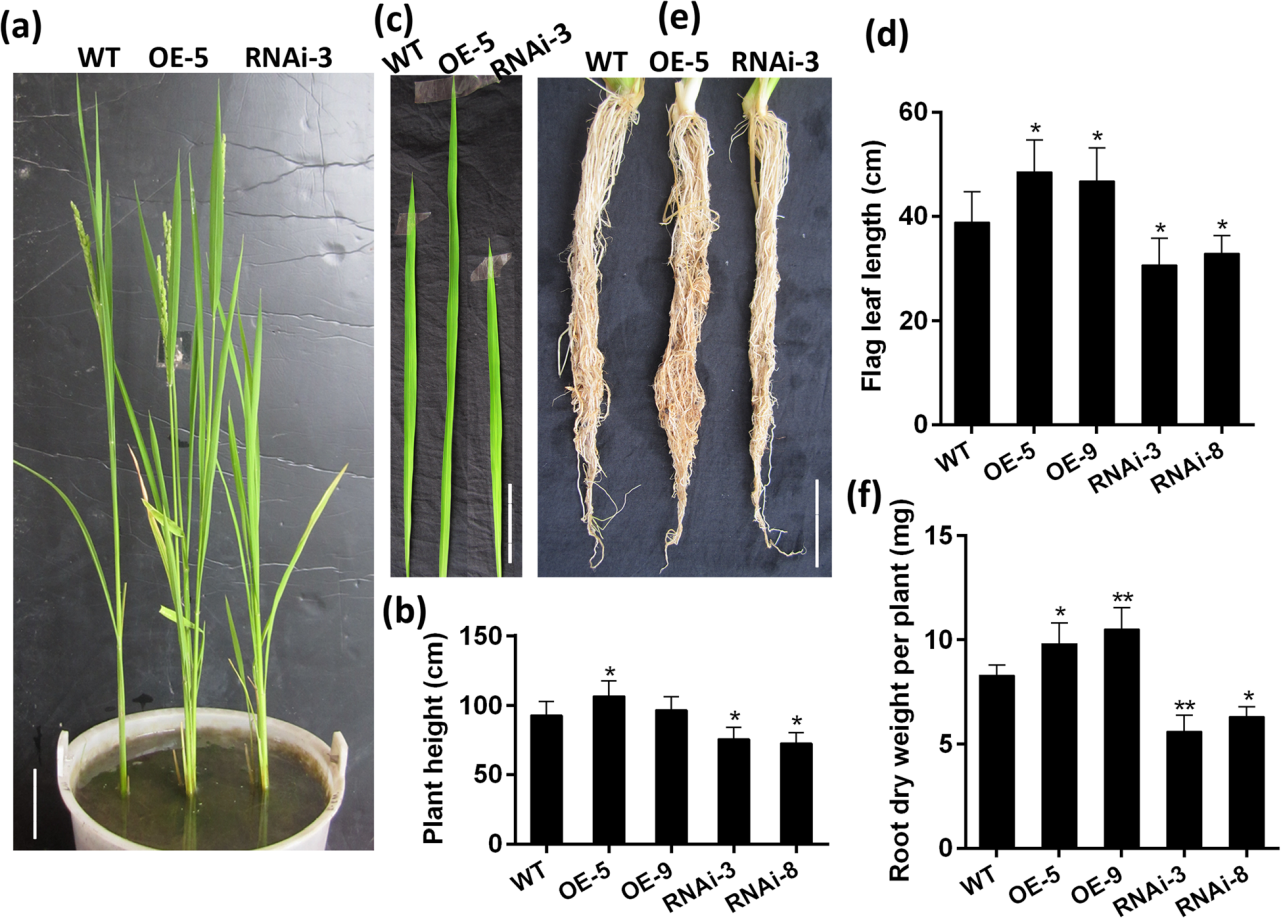
Plant morphology and root system architecture of pot-grown *OsFPFL4* transgenic plants at maturity. **a** Plant morphology of two-month-old WT and *OsFPFL4* transgenic plants. Scale bars, 10 cm. **b** Measurement of plant height. **c** Flag leaf of two-month-old WT and *OsFPFL4* transgenic plants. Scale bars, 2 cm. **d** Measurement of flag leaf length. **e** Root system architecture of two-month-old WT and *OsFPFL4* transgenic plants. Scale bars, 6 cm. **f** Dry weight of root system per plant. WT, wild type. OE-5 and OE-9, *OsFPFL4* overexpression transgenic lines. RNAi-3 and RNAi-8, *OsFPFL4*-RNAi transgenic lines. Data are means ± SE (*n* = 10). The significant difference between *OsFPFL4* transgenic lines and wild type was determined by Student’s *t*-test. ^*^p < 0.05, ^**^p < 0.01

To investigate the role of *OsFPFL4* in plant growth and development in rice, we generated transgenic plants by overexpression or RNA interference (RNAi) of *OsFPFL4*, respectively (Fig. 3a and b). The root system of both *OsFPFL4*-overexpression (OE-5 and OE-9) and -RNAi transgenic (RNAi-3 and RNAi-8) lines was changed greatly, compared with their wild-type counterparts after grown for 7 days in 1/2 MS agar medium (Fig. 3a). Rice plants overexpressing *OsFPFL4* exhibited promotion of the shoot growth as well as lateral root and adventitious root formation, whereas RNAi lines had the phenotype of repressed root and shoot growth (Fig. 3a). Compared to wild type, both *OsFPFL4*-overexpression and -RNAi seedlings showed markedly reduced primary root length (Fig. 3d). The shoot length, lateral root density and adventitious root number in overexpression plants were greatly increased, but the counterparts of RNAi plants were reduced significantly (Fig. 3c, e and f). In addition, up- or down-regulation of *OsFPFL4* reduced the average length of adventitious root (Fig. 3g). Although the length of lateral root was not quantified, it was clearly observed to be reduced in RNAi lines compared with that of wild type (Fig. 3h). It is notable that overexpression lines exhibited helix primary root (Fig. 3a). In comparison with wild-type plants grown for 60 days, the plants overexpressing *OsFPFL4* displayed better performance with increased plant height and flag leaf length, whereas the growth of RNAi plants was remarkably inhibited (Fig. 4a-d). There seems to be no significant difference in the length of root system between wild type and transgenic lines grown in soil for 60 days, but the overexpression plants had more branched root system than wild type and RNAi lines (Fig. 4e). Accordingly, the biomass of root system was significantly enhanced in overexpression plants, whereas reduced greatly in RNAi plants, compared that of wild type (Fig. 4f), which might be caused by the difference of adventitious root number and lateral root density. Together, these results indicate that *OsFPFL4* plays a key role in the root growth and development in rice.

### Abnormal Expression of *OsFPFL4* Affects the Development of Anther and Pollen

**Fig. 5:**
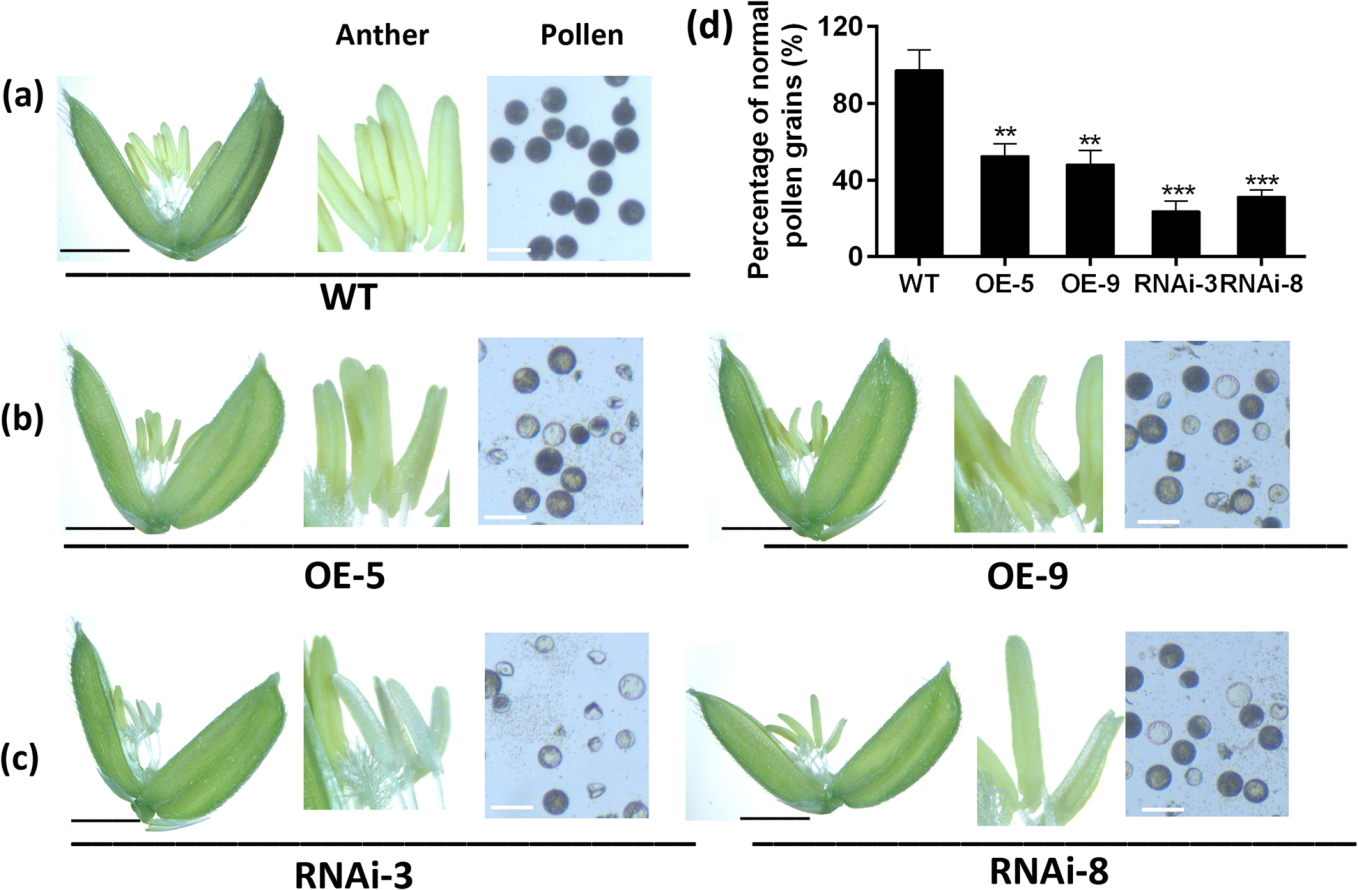
Phenotypes of the stamen and pollen in *OsFPFL4* transgenic plants. **a** The development of stamen and pollen in WT. **b** The development of stamen and pollen in *OsFPFL4* overepression plants. **c** The development of stamen and pollen in *OsFPFL4* RNAi plants. Scale bars in spikelet are 2 mm. Scale bars in grain pollen are 200 μm. **d** Percentage of normal pollen. Mature pollen grains were stained with I_2_-KI on day 3 post-flowering. WT, wild type. OE-5 and OE-9, *OsFPFL4* overexpression transgenic lines. RNAi-3 and RNAi-8, *OsFPFL4*-RNAi transgenic lines. Data are means ± SE (*n* = 10). The significant difference between *OsFPFL4* transgenic lines and wild type was determined by Student’s *t*-test. ^*^p < 0.05, ^**^p < 0.01 or ^***^p < 0.001

Considering that *OsFPFL4* is preferentially expressed in inflorescence at heading stage (Fig. 2a), we then explored the role of *OsFPFL4* in the control of flowering time and flower organ development. As shown in Fig. 4a, overexpression of *OsFPFL4* did not obviously lead to early flowering in rice; however, RNAi lines exhibited apparently delayed flowering. As to flower organ development, there seemed to be no visible difference in the pistil between wild type and transgenic plants; however, abnormal expression of *OsFPFL4* caused defective development of stamens (Fig. 5). The yellow and plump anthers appeared in wild-type plants at the flowering stage (Fig. 5a). Conversely, stamens of the *OsFPFL4*-overexpression or -RNAi plants showed obvious defects and were also smaller than the counterparts of wild type (Fig. 5b and c). Compared to wild type, up-regulation of *OsFPFL4* resulted in slightly shrunken anthers, whereas down-regulation of *OsFPFL4* led to white and shrunken anthers more severely (Fig. 5a-c). We further observed the pollen development on day 5 after flowering. In comparison with wild type, a great number of abnormal pollens were observed in *OsFPFL4*-overexpression or RNAi plants, and the fertility of mature pollen in the RNAi lines seemed to be damaged more severely, indicated by 1% I_2_-KI staining (Fig. 5a-c). Mature pollens of wild type were black and round in I_2_-KI, while pollens from *OsFPFL4*-overexpression or RNAi plants at the same development period were grey and shriveled, or even broken (Fig. 5a-c), suggesting the pollen vitality might be severely impaired. Further investigation showed that the ratio of normal pollen of transgenic lines was remarkably reduced in overexpression or RNAi lines, compared to that of wild type (Fig. 5d). Together, these results indicate that normal expression of *OsFPFL4* is required for the development of stamen and pollen in rice, implying its crucial roles in the grain yield.

### *OsFPFL4* is Involved in the Root Development by Modulating Auxin Homeostasis

**Fig. 6:**
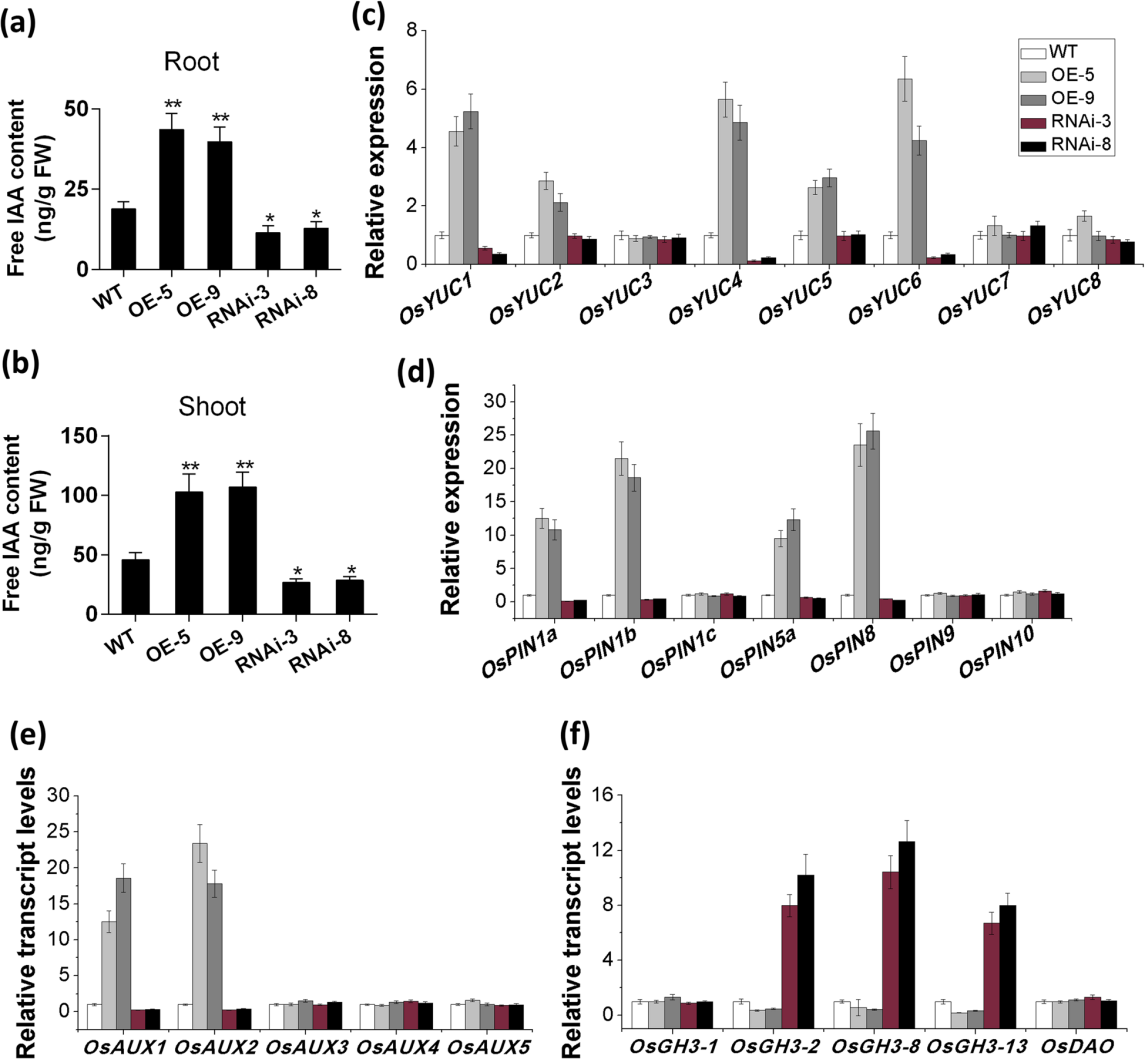
*OsFPFL4* increases auxin accumulation in the shoots and roots via coordinated auxin biosynthesis, transport and degradation. **a** and **b** Quantification of auxin content in the 9-day-old shoots and roots, respectively. Data are means ± SE (*n* = 10). Relative transcription levels of the genes involved in auxin biosynthesis (**c**), transport (**d** and **e**), and degradation (**f**). Data are means ± SE (*n* = 3). Three replica experiments were performed. WT, wild type. OE-5 and OE-9, *OsFPFL4* overexpression transgenic lines. RNAi-3 and RNAi-8, *OsFPFL4*-RNAi transgenic lines. The significant difference between *OsFPFL4* transgenic lines and wild type was determined by Student’s *t*-test. ^*^p < 0.05, ^**^p < 0.01. All data displayed as a mean ± SD. Three independent experiments were performed (*n* = 30 plants per genotype in each independent experiment)

It has been reported that the primary root and lateral root respond to auxin differently, and exogenous auxin can promote the lateral root development but reduce primary root elongation (Marin et al. 2010; Yoon et al. 2014). The phenotypes of increased lateral root density and inhibited primary root elongation were observed in *OsFPFL4* overexpression plants (Fig. 3d and e), implying that endogenous auxin accumulation or polar auxin transport might be altered in *OsFPFL4* transgenic plants. To determine whether the alteration of lateral root density and primary root length in transgenic plants was associated with endogenous auxin levels in roots, the IAA content was investigated in the overexpression, RNAi and wild-type seedlings by HPLC-MS-MS analysis. As shown in Fig. 6a, IAA content was significantly increased in roots of overexpression plants, whereas drastically reduced in RNAi plants, compared to that in wild type. In fact, enhanced auxin accumulation was also found in shoots of overexpression plants, whereas reduced auxin content was detected in RNAi shoots (Fig. 6b). Auxin levels in the roots are determined by local auxin biosynthesis, polar auxin transport, auxin breakdown, and/or auxin conjugation. We then investigated the transcript levels of auxin biosynthesis genes (*OsYUCs*), polar auxin transport genes (*OsPINs* and *OsAUX/LAXs*), and auxin inactivation genes (*OsDAO* and *OsGH3s*) in the roots. The results showed that *OsYUC1*, and *OsYUC4* and *OsYUC6* were found to be greatly up-regulated in overexpressing plants, whereas down-regulated in RNAi plants (Fig. 6c). As to *OsPIN*s and *OsAUX/LAXs*, the transcript levels of *OsPIN1a*, *OsPIN1b*, *OsPIN5a*, *OsPIN8*, *OsAUX1* and *OsAUX2* were found to be drastically increased in overexpressing plants, but reduced in RNAi plants (Fig. 6d and e). Other genes involved in auxin biosynthesis and polar auxin transport did not show significant difference in transcript levels between transgenic lines and wild type (Fig. 6d and e). Transcription of *OsGH3*–*2*, *OsGH3*–*8* and *OsGH3*–*13* was greatly increased in RNAi plants, but there is no difference in *OsDAO* expression between transgenic lines and wild type (Fig. 6f).

### Abnormal Expression of *OsFPFL4* Enhances ROS Levels

**Fig. 7:**
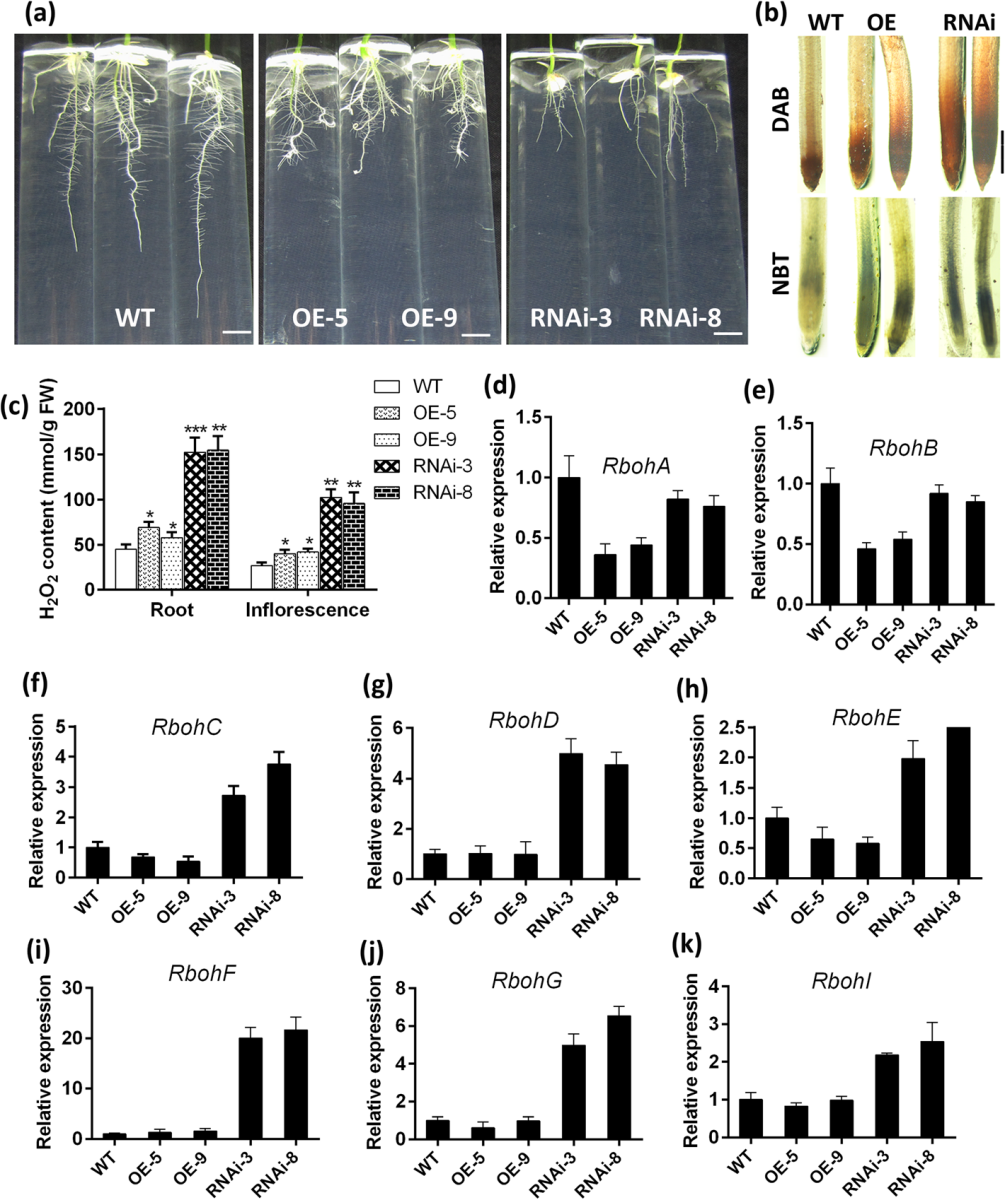
*OsFPFL4* alters ROS accumulation in the root and inflorescence through affecting the expression of ROS-producer *Rbohs*. **a** Root system architecture of 7-day-old seedling in 1/2 MS medium. Scale bars, 1 cm. **b** NBT and DAB staining for O_2_^−^ and H_2_O_2_, respectively, in the root tips of wild type and *OsFPFL4* transgenic lines. Scale bars, 1 mm. **c** Quantification of H_2_O_2_ content in the roots of wild type and *OsFPFL4* transgenic plants. Data are means ± SE (*n* = 10). **d**-**k**Transcript levels of *OsRbohs* in the roots of wild type and *OsFPFL4* transgenic plants by qPCR analysis. Data are means ± SE (*n* = 3). Three replica experiments were performed. WT, wild type. OE-5 and OE-9, *OsFPFL4* overexpression transgenic lines. RNAi-3 and RNAi-8, *OsFPFL4*-RNAi transgenic lines. Data are means ± SE (*n* = 10). The significant difference between *OsFPFL4* transgenic lines and wild type was determined by Student’s *t*-test. ^*^p < 0.05, ^**^p < 0.01 or ^***^p < 0.001

The action of auxin in the regulation of root development is closely associated with ROS (Zhang et al. 2014a; Xia et al. 2015). To test whether the roots of *OsFPFL4* transgenic lines have altered ROS levels, we used the ROS-reactive dyes DAB and NBT to detect the levels of H_2_O_2_ and O_2_^−^, respectively, in the primary root tip (Fig. 7a). As shown in Fig. 7b, the root tips of both *OsFPFL4*-overexpression and -RNAi lines had the stronger staining than that of wild type, and *OsFPFL4*-RNAi roots had the strongest staining. Compared to the strong staining just centralized in the first 1 mm of the root tip in wild type, the staining intensity continued to be strong in the differentiation zone and elongation zone of the root tip of *OsFPFL4*-RNAi plants (Fig. 7b). Investigation of H_2_O_2_ content showed that its accumulation was slightly increased in *OsFPFL4*-overexpression roots but drastically enhanced in RNAi roots (Fig. 7c). ROS accumulation was also enhanced in inflorescence in transgenic plants (Fig. 7c). The apoplastic ROS are mainly produced by plasma membrane localized NADPH oxidases (Kadota et al. 2015). Thus, we further evaluated the transcription of ROS producers, *Rbohs*. As shown in Fig. 7d-k, the transcript levels of most of *OsRbohs* were clearly reduced in *OsFPFL4* overexpression roots, but significantly enhanced in *OsFPFL4*-RNAi roots, compared to that in wild type. These results suggest that *OsFPFL4* might alter the expression of these ROS-producers to affect ROS levels.

**Fig. 8:**
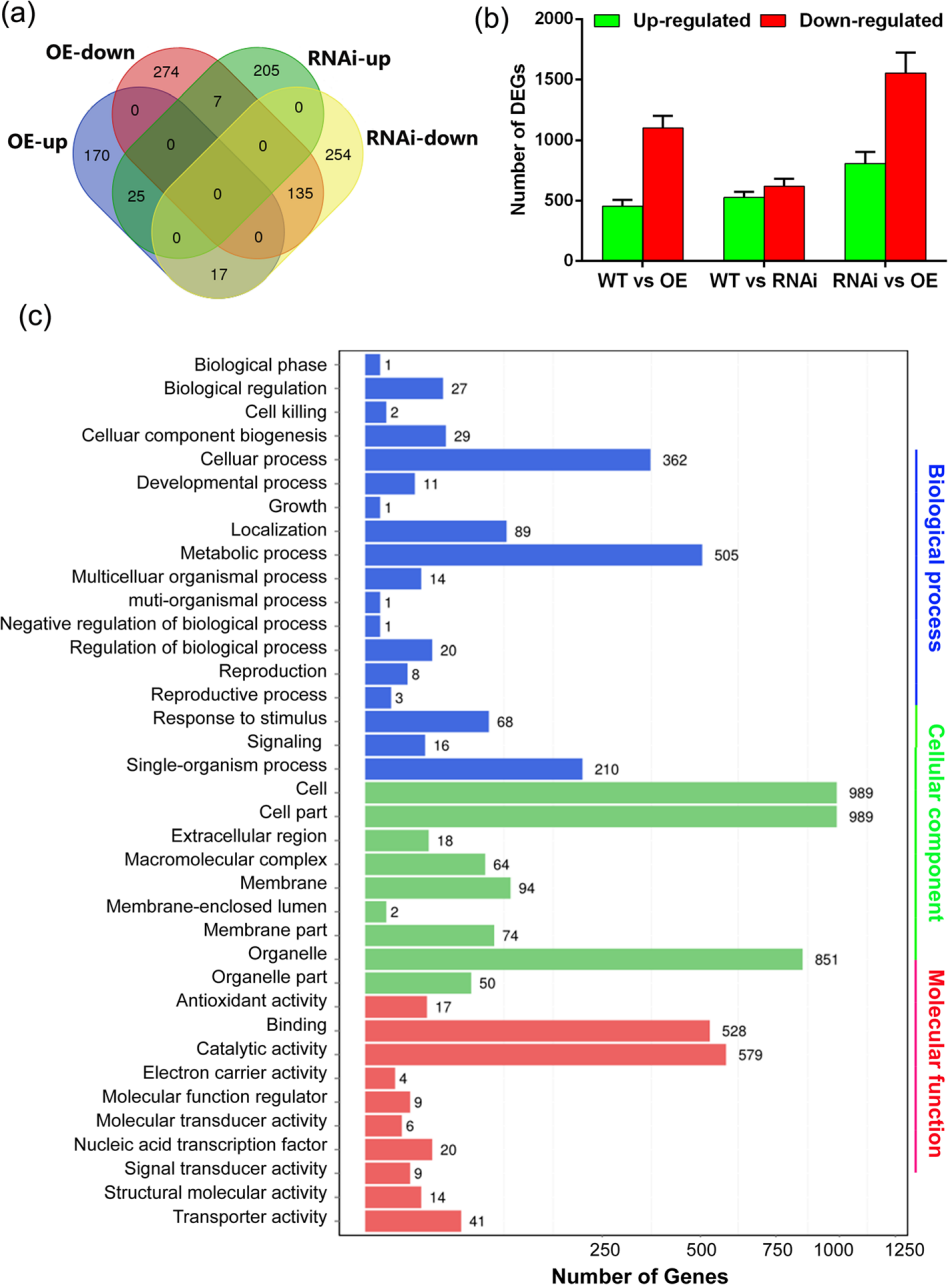
Analysis of RNA-Seq data of wild type, *OsFPFL4* overexpression and -RNAi transgenic lines. **a** Venn’ plot of differentially expressed genes (DEGs) between wild type and *OsFPFL4* transgenic lines. **b** The number of DEGS between wild type and *OsFPFL4* transgenic lines. **c** Functional categorization of the DEGs between wild type and *OsFPFL4* transgenic lines based on Gene Ontology (GO) annotation. Seedlings were grown in 1/2 MS medium for 7 days, and three biological replicates were used. LOC_Os01g27340, LOC_Os10g38720, LOC_Os03g17480, LOC_Os03g57200, LOC_Os10g38740, LOC_Os09g20220 are genes for glutathione S-transferases; LOC_Os07g01420, LOC_Os01g18950, LOC_Os07g44480, LOC_Os01g18970, LOC_Os10g39160, LOC_Os07g44460, LOC_Os07g31610, LOC_Os03g25320, LOC_Os06g16350, LOC_Os03g25280 are genes for peroxidases

**Fig. 9:**
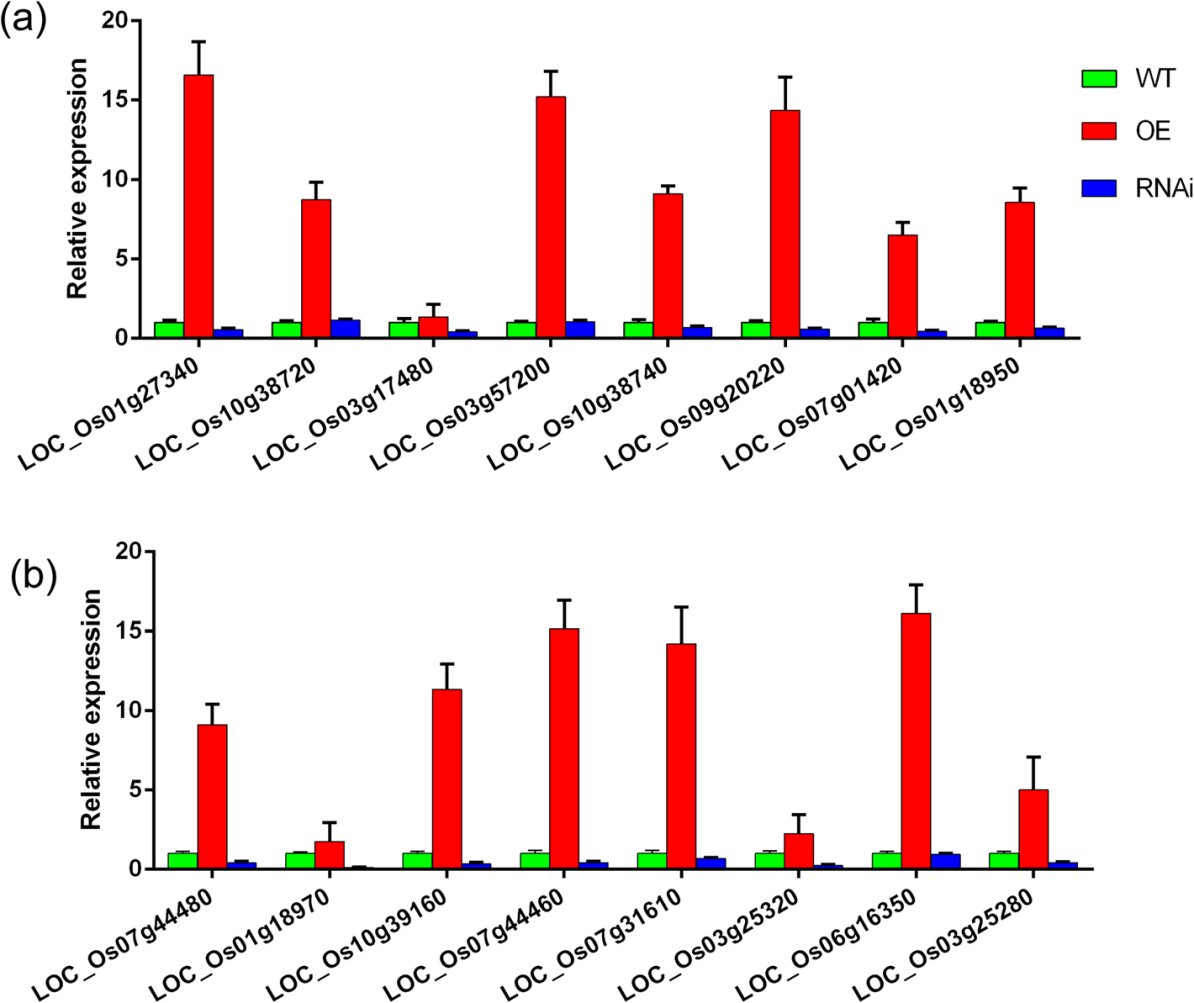
Transcript levels of ROS-scavengers in wild and *OsFPFL4* transgenic plants by quantitative PCR analysis. Seedlings were grown in 1/2 MS medium for 7 days. The data represent the means (± SE) of three biological replicates. Three replica experiments were performed. WT, wild type. OE-5 and OE-9, *OsFPFL4* overexpression transgenic lines. RNAi-3 and RNAi-8, *OsFPFL4*-RNAi transgenic lines

In order to understand the functions of *OsFPFL4* in the growth and development in rice plants in depth, global gene expression analysis of *OsFPFL4* transgenic plants and wild type by RNA sequencing (RNA-Seq) was performed. As shown in Fig. 8a and b, there were a total of 2362 differentially expressed genes (DEGs) (*P* < 0.05) between *OsFPFL4* overexpression and RNAi lines. Go Analysis showed that these DEGs are involved in various biological process such as growth and development, response to stimuli, signal transduction and cell apoptosis (Fig. 8c). In plants, ROS homeostasis is strictly controlled by a delicate balance between ROS-producing and -scavenging enzymes (Qi et al. 2017). To our interesting, RNA-Seq analysis showed that the expression levels of multiple genes for ROS-scavenging enzyme glutathione *S*-transferase were increased in *OsFPFL4* overexpression plants but reduced in RNAi lines (Additional file 2: Table S2). The transcription of many peroxidases, which are considered as bifunctional enzymes that can scavenge H_2_O_2_ but also produce ROS, were also altered in transgenic lines (Additional file 2: Table S2). To validate the expression profiles obtained by RNA-Seq analysis, qPCR analysis was performed for these genes in Additional file 2: Table S2. As expected, qPCR analysis displayed similar patterns as the RNA-Seq data, despite some of quantitative differences in expression levels (Fig. 9). The results suggest that abnormal expression of *OsFPFL4* might affect ROS levels by changing the transcription of genes for ROS-producing and -scavenging enzymes in rice plants.

## Discussion

*OsFPFL4* belongs to a small protein family that have no introns in their genomic sequences and was involved in the genetic control of flowering time in plant, denoted as *FPF1* (Kania et al. 1997; Melzer et al. 1999; Ge et al. 2004; Wang et al. 2014). *MuFPF1* was firstly studied as a flowering-promoting factor in mustard (Melzer et al. 1990), and its overexpression in Arabidopsis led to early flowering (Kania et al. 1997). *AtFPF1* was proved to modulate flowering time via the GA-dependent signaling pathway in Arabidopsis (Melzer et al. 1999), and overexpression of *AtFPF1* confers promotion of flowering time as well as formation of adventitious root in rice (Xu et al. 2005). *OsRAA1*, a homologue of *FPF1*, shared a 58% sequence homology of amino acids with *AtFPF1*, and its overexpression caused pleiotropic phenotypes in transgenic rice plants, including altered leaf shape, flower and root development as well as root response to gravity (Ge et al. 2004). It is notable that, different from other *FPF1*s, overexpression of *OsRAA1* did not promote flowering time, but caused abnormal florets with longer filaments and shrunken anthers (Ge et al. 2004). In our study, overexpression of *OsFPFL4* did not obviously lead to early flowering in rice; however, RNAi lines exhibited delayed flowering (Fig. 4a), and *OsFPFL4* transgenic lines had normal florets (Fig. 5a-c). There are a few genes involved in the root as well as flower development. MADS-box genes that are known to regulate the network of flower development also work in signal transduction in root development (Zhang and Forde 1998; Yu et al. 2017; Zhang et al. 2018). Similar to *OsRAA1*, *OsFPFL4* was also indicated to modulate root development. Overexpression of *OsFPF4* significantly reduced the primary root length, but greatly increased the lateral root density and adventitious root number (Fig. 3e and f), suggesting that there is similarity in the genetic control of flowering and root development between *OsFPFL4* and *OsRAA1*.

The lateral root density were remarkably increased in *OsFPFL4* overexpression lines, whereas reduced in RNAi lines (Fig. 3e), indicating that *OsFPFL4* promotes formation of the lateral root. Increased auxin accumulation and transport can enhance lateral root formation (Peret et al. 2009; Zhao et al. 2015). In this study, we showed that *OsFPFL4* promoted lateral root development by modulating auxin accumulation in the root. Firstly, the transcript levels of *OsFPFL4* were positively correlated with the lateral root number (Fig. 3b and e), a process that depends on auxin-mediated establishment. Then, alteration in root auxin levels in *OsFPFL4*-overexpressing and -RNAi lines were clearly confirmed by the measurement of endogenous IAA content (Fig. 6a). We hypothesize that *OsFPFL4* influenced auxin homeostasis in the root by increasing polar auxin transport and/or local auxin biosynthesis, and/or reducing auxin degradation/conjugation. *YUCs* are evidently key genes for auxin biosynthesis (Zhao et al. 2001), and overexpression of *YUCs* led to the overproduction of auxin, whereas disruption of *YUC*s caused developmental defects in roots (Peret et al. 2009). Influx and efflux transporters mediated polar auxin transport, which controlled plant root development (Blilou et al. 2005; Peret et al. 2012). *AUX/LAX* loss-of-function mutations led to reduced lateral root formation by affecting lateral root initiation and/or emergence (Marchant et al. 2002; Swarup et al. 2008; Zhao et al. 2015). As a group of early auxin-responsive genes, the *GH3* family encodes IAA-amido synthetases that prevent free IAA accumulation (Du et al. 2012). In our study, we found that *OsFPFL4* changed the transcript levels of *OsYUCs*, *OsPINs/OsAUXs*, and *OsGH3s* (Fig. 6c-f), which could be one of the reasons why auxin content was altered in *OsFPFL4* transgenic plants (Fig. 6a and b). Therefore, our data support that *OsFPFL4* modulates lateral root formation in rice by altering auxin accumulation in the root.

It has been reported that the ROS and auxin pathways can extensively impact each other (Kwak et al. 2006). Auxin-induced ROS as signals are directly involved in cell-wall loosening and cell elongation as well as auxin-mediated developmental processes (Schopfer 2001; Xia et al. 2015), and cellular redox status is an intrinsic regulator of the plant cell cycle (Diaz Vivancos et al. 2010). Meanwhile, H_2_O_2_ can regulate the root system architecture by modulating the polar transport and redistribution of auxin (Su et al. 2016), and asymmetric ROS accumulation mediates auxin-regulated root gravitropism (Joo et al. 2001). More specifically, root elongation is reduced by ROS via enhancing *Rbohs* expression, whereas silencing of *RbohC* accelerates root elongation (Zhang et al. 2014a). Actually, increased ROS may alter auxin signalling through oxidative inactivation or degradation of auxin, and also by the decreased expression of genes involved in auxin signalling and polar auxin transport (Blomster et al. 2011; Peer et al. 2013). For example, when plants are exposed to environmental stresses, ROS can attenuate auxin signalling, leading to altered plant growth and acclimation. In our study, *OsFPFL4* overexpression lines, which had more free IAA than wild type, exhibited slightly increased ROS accumulation (Fig. 6a and b; Fig. 7b and c). Although the mRNA levels of *Rboh*s were reduced in overexpression lines, the expression of multiple genes encoding PERs, which also catalyze ROS production, was increased (Fig. 7d-k; Additional file 2: Table S2). In RNAi lines that had less free IAA accumulation, ROS levels were significantly increased, accompanied by elevated expression of *Rboh*s (Figs. 6a, 7b-k). The mechanism of auxin-induced ROS production has been pursued. Recently, great progress has been achieved in the molecular link between auxin- and ROS-mediated developmental program. In Arabidopsis, the bHLH transcription factor RSL4 (ROOT HAIR DEFECTIVE 6 LIKE 4) was activated by ARFs which binded to *RSL4* promoter on Aux-RE sites (Pires et al. 2013; Mangano et al. 2017). Auxin-ARF activation of RSL4 promoted ROS production by directly regulating the expression of *RbohC*, *RbohJ* and several *PERs* (Hwang et al. 2017; Mangano et al. 2017), suggesting that auxin-induced ROS-mediated developmental program is fine-tuned by the master regulator, RSL4. To elucidate the underlying mechanism that OsFPFL4 orchestrates root and flower development in response to these key developmental signals, ROS and auxin, it is crucial to find transcription factors that function like RSL4 in rice.

## Conclusions

**Fig. 10:**
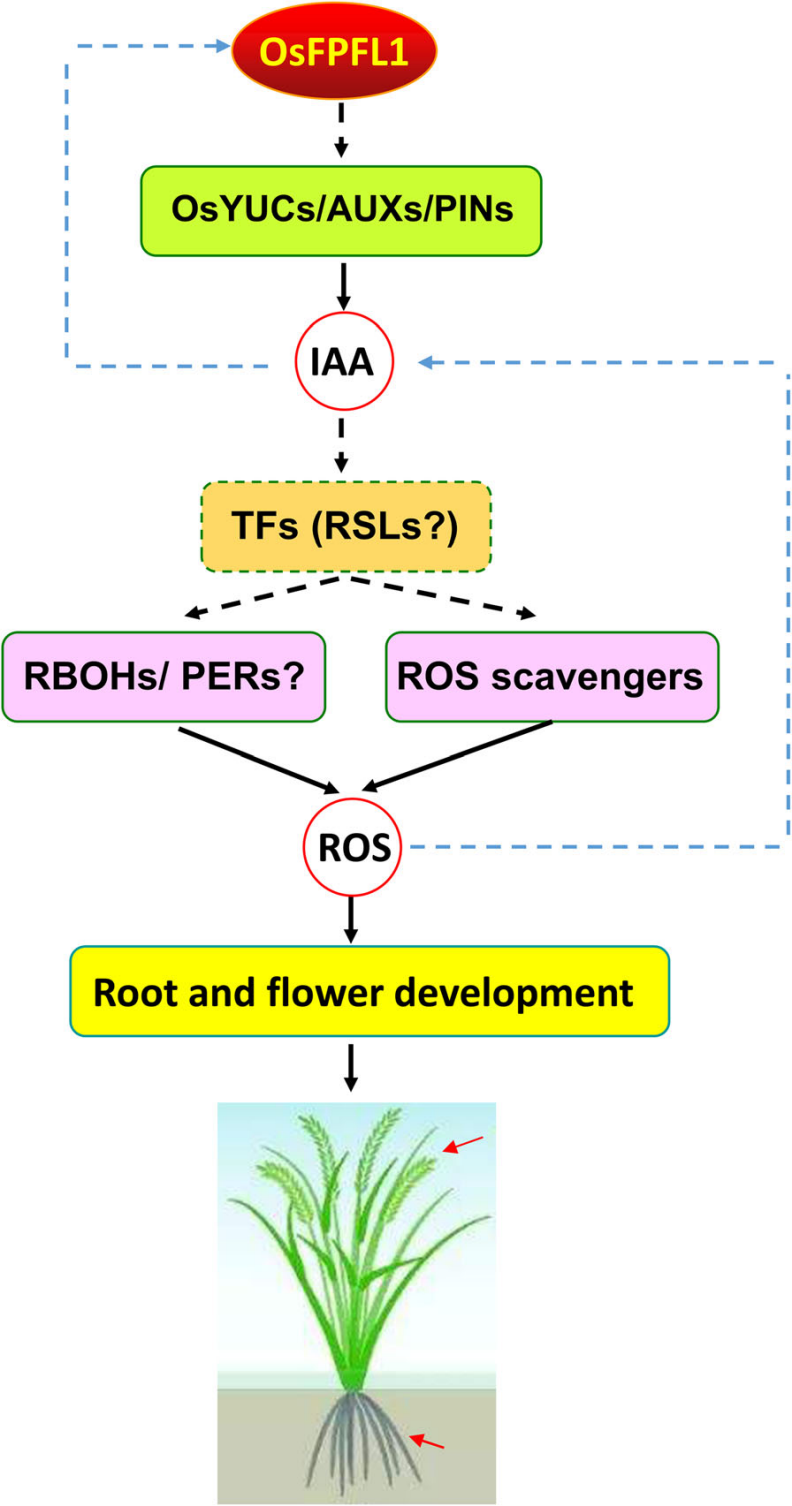
Working model describing *OsFPFL4* controls the root and flower development via affecting auxin as well as ROS homeostasis. *OsFPFL4* controls auxin as well as ROS homeostasis via affecting the transcription of genes for auxin biosynthesis and/or transport, and ROS producing and/or scavenging. Auxin-mediated ROS production might play a role in regulating redox status by RSL4 analogs. Alternatively, ROS control plant growth and development potentially through regulating auxin homeostasis

Collectively, our results showed that *OsFPFL4* is invovled in the regulation of root and flower development through controlling auxin as well as ROS homeostasis. Specifically, auxin-mediated ROS production might play a role in regulating redox status, which, in turn, modulates auxin homeostasis and signaling, to control plant growth and development (Fig. 10). Thus, ROS production and associated redox processing are an integral part of hormone regulation and function in the control of plant growth and development.

## Materials and Methods

### Plant Materials and Growth Conditions

The *japonica* rice (*Oryza sativa* L) variety ‘Nipponbare’ was used for physiological experiments and genetic transformation in this study. For phenotypic observations, rice seeds were surface sterilized with ethanol (75%, v/v) and diluted NaClO (1:3, v/v), followed by thorough rinse with sterilized water, and then were germinated in 1/2 MS medium (Murashige and Skoog 1962). Seedlings were grown in a growth chamber at 30 °C with a 14-h photoperiod and a light intensity of 300 μmol photons m^− 2^ s^− 1^. Relative humidity was controlled at approximately 60%.

### Phytohormone and Abiotic Stress Treatments

Investigation of the responses to phytohormone and abiotic stress treatments was performed according to the described methods (Puig et al. 2013), with minor modifications. Ten-day-old seedlings were treated with 20 μM IAA, 200 mM NaCl, 100 mM mannitol, 20% PEG6000, 42 °C and 4 °C, respectively. Roots of these seedlings were sampled to evaluate the expression of *OsFPFL4*.

### Vector Construction and Generation of Transgenic Plants

For the overexpression construct, the full-length cDNA of *OsFPFL4* was amplified and cloned into the modified pCAMBIA1301 vector via *Bam*HI and *Pst*I and driven by *35S* promoter. For the RNA-silencing construct, a cDNA fragment of *OsFPFL4* was cloned into the pENTR/D-TOPO vector (Invitrogen) to get the entry clone pENTR/*OsFPFL4*. The final RNA-silencing vector, *OsFPFL4-*RNAi, was generated by a clonase reaction (Invitrogen) between pENTR/*OsFPFL4* and the vector pANDA (Miki and Shimamoto 2004). The constructs were transformed into rice calli by using *Agrobacterium tumefaciens*-mediated transformation as previously described (Ozawa and Takaiwa 2010). Then the transgenic lines were screened based on the hygromycin resistance, GUS staining and expression levels of *OsFPFL4*. Primers for vector construction are listed in Additional file 1: Table S1.

### Morphological Characterization and Quantification of Root System Traits

Root morphology was examined in seedlings grown on 1/2 MS agar medium. All visible lateral roots originating from the primary root were counted. Adventitious root length was calculated as the average of the three longest adventitious roots (Yan et al. 2014).

### RNA Extraction and Quantitative Real-Time PCR

Total RNA was extracted from different tissues using TRIzol reagent and used for reverse-transcription. Quantitative real-time PCR analysis of the targeted genes was then performed, using rice *β*-*actin* gene as the internal control. Primer sequences for quantitative real-time PCR analysis are given in Additional file 1: Table S1. Three replica experiments were performed for each analysis.

### Determination of IAA Content

IAA content was determined on a high performance liquid chromatography tandem mass spectrometer (HPLC-MS-MS) instrument (AB Sciex QTRAP®6500, Agilent Technologies) according to the protocol described previously (You et al. 2016). Fresh shoot or root samples (300 mg) from 10-day-old seedlings grown on 1/2 MS medium were collected and used for IAA content measurement.

### ROS Assays

We used nitroblue tetrazolium (NBT) staining to detect O_2_^−^, and 3, 3′-diaminobenzidine (DAB) staining for H_2_O_2_, as described previously (Zhang et al. 2014a). H_2_O_2_ quantification was performed according to the method as described previously (Zhang et al. 2014b).

### Pollen Viability Assay

Evaluation of pollen grain viability was performed as previously described (Shi et al. 2015). Anthers from mature spikelets were crushed and stained in 1% I_2_-KI solution for 5 min, and then the pollens were observed and photographed under a light microscope using bright-field illumination. The frequency of darkly stained pollen grains was determined from at least 10 plants of each line.

## Supplementary information

**Additional file 1: Table S1.** Primer sequences used in this study.

**Additional file 2: Table S2.** DEGs (differentially expressed genes) involved in ROS homeostasis in *OsFPFL4* transgenic plants.

**Additional file 3: Figure S1.** FPF1-like proteins in rice.

## Data Availability

The datasets used or analysed in this study are included in the article and its additional files.

## Abbreviations

DAB: 3, 3′-diaminobenzidine
NBT: Nitroblue tetrazolium
ROS: Reactive oxygen species
