# Supplementary material for: OsFPFL4 is Involved in the Root and Flower Development by Affecting Auxin Levels and ROS Accumulation in Rice (Oryza sativa)
Source: Rice (N Y). 2020 Jan 7;13:2. doi: 10.1186/s12284-019-0364-0 (PMC6946790; doi:10.1186/s12284-019-0364-0)
Supplement: Supplementary file 1 — Additional file 1: Table S1. Primer sequences used in this study. [file 12284_2019_364_MOESM1_ESM.doc]

Table S1 Primer sequences used in this study

| **Primers for qRT-PCR** | | | |
| --- | --- | --- | --- |
| Name | Sequences(5’ -3’) | | Experiments |
| OsMADS23 –F/R | TGGTCCAAATCTTATTGGAGTG / TTGATGGACAAGGCTGACTTT | | qRT-PCR |
| OsRbohA –F/R | gtcttatgcagtcatgaatgtaca / gaataatatacagttaattagcct | | qRT-PCR |
| OsRbohB –F/R | cctagtggaagaagctgtgct / cactatgaaagggaacatcacaa | | qRT-PCR |
| OsRbohC –F/R | tgttttagggatggttttacac / tgtacagacagaaggttaacgt | | qRT-PCR |
| OsRbohD –F/R | gaccagaccaggaaaaaaacaccaa/acacagaaagagttgctaaccgatg | | qRT-PCR |
| OsRbohE –F/R | catcgtgcatagattctgga / catgcattcccactgttcca | | qRT-PCR |
| OsRbohF –F/R | tcgtctatcatagatatacatg / cgtgtactttggtgacctcag | | qRT-PCR |
| OsRbohG –F/R | aagcgttgctaattttcgctat / gagaggatgtttttttgaacgg | | qRT-PCR |
| OsRbohI–F/R | tggccagataatttcatcggtt / gctactctaagtattacaaagta | | qRT-PCR |
| OsCu/Zn-SOD-F/R | CTTGCGGGATCATCGGACTT / GAGCTCGTAGAGTTTCAGGCT | | qRT-PCR |
| OsMn-SOD -F/R | GCCAGACTACCTGAGCAACA/ CGTTGCCTTCAGACAACATCA | | qRT-PCR |
| OsAPX1-F/R | CCAAGGGTTCTGACCACCTA/ CAGTTCGGAGAGCTTGAGGT | | qRT-PCR |
| β-actin-F/R | AGGAAGGCTGGAAGAGGACC/ CGGGAAATTGTGAGGGACAT | | qRT-PCR |
| OsZIP23- F/R | GGAGCTGAACGATGAACTCCAG/ TCGGCTCATTCTCTCTAGAACCTC | | qRT-PCR |
| OsZIP46- F/R | GAACACTGACTGGTCCATGCTG/ GAGAGAAGCAACTCTGAAGCTGAG | | qRT-PCR |
| **Primers for generating DNA vector** (The underline showed the restriction enzyme sites. F, forward; R, reverse) | | | |
| Name | Sequences(5’ -3’) | Experiments | |
| OsFPFL1- F | CGGGATCCAACATATATATATTATATAT | PCAMBIA1301, overexpression | |
| OsFPFL1- R | AACTGCAG AGCATAGACATCGATGCGATCAT | PCAMBIA1301, overexpression | |
| OsFPFL1 -D-topo-F | AGTTCCACCAGCGCTCCACCGTAGAA | D-topo, RNAi | |
| OsFPFL1 -D-topo-R | ATATGATATACAATCATTCATTA | D-topo, RNAi | |
| *Hygromycin-*F | ACGGTGTCGTCCATCACAGTTTGCC | Identification of positive transfomants | |
| *Hygromycin-*R | TTCCGGAAGTGCTTGACATTGGGGA | Identification of positive transfomants | |
| *Ubiquitin-*F | CGAGTCTAACGGACACCAC | Identification of positive transfomants | |
| *Ubiquitin-*R | /AAAGATGACCCGACAAA | Identification of positive transfomants | |
