# Supplementary material for: OsFPFL4 is Involved in the Root and Flower Development by Affecting Auxin Levels and ROS Accumulation in Rice (Oryza sativa)
Source: Rice (N Y). 2020 Jan 7;13:2. doi: 10.1186/s12284-019-0364-0 (PMC6946790; doi:10.1186/s12284-019-0364-0)
Supplement: Supplementary file 2 — Additional file 2: Table S2. DEGs (differentially expressed genes) involved in ROS homeostasis in OsFPFL4 transgenic plants. [file 12284_2019_364_MOESM2_ESM.docx]

Table S2 DEGs might be involved in balancing ROS homeostasis in *OsFPFL1* transgenic lines by RNA-seq analysis

| No. | Gene ID | Annotation |
| --- | --- | --- |
| 1 | LOC_Os01g27340 | glutathione S-transferase, putative |
| 2 | LOC_Os10g38720 | glutathione S-transferase |
| 3 | LOC_Os03g17480 | glutathione S-transferase |
| 4 | LOC_Os03g57200 | glutathione S-transferase, putative |
| 5 | LOC_Os10g38740 | glutathione S-transferase |
| 6 | LOC_Os09g20220 | glutathione S-transferase, putative |
| 7 | LOC_Os07g01420 | peroxidase precursor |
| 8 | LOC_Os01g18950 | peroxidase precursor |
| 9 | LOC_Os07g44480 | Peroxidase, putative |
| 10 | LOC_Os01g18970 | peroxidase, putative |
| 11 | LOC_Os10g39160 | peroxidase, putative |
| 12 | LOC_Os07g44460 | peroxidase |
| 13 | LOC_Os07g31610 | peroxidase, putative |
| 14 | LOC_Os03g25320 | peroxidase, putative |
| 15 | LOC_Os06g16350 | peroxidase |
| 16 | LOC_Os03g25280 | peroxidase, putative |

DEGs: differentially expressed genes.
