# Supplementary figures and images for: OsFPFL4 is Involved in the Root and Flower Development by Affecting Auxin Levels and ROS Accumulation in Rice (Oryza sativa)
Source: Rice (N Y). 2020 Jan 7;13:2. doi: 10.1186/s12284-019-0364-0 (PMC6946790; doi:10.1186/s12284-019-0364-0)

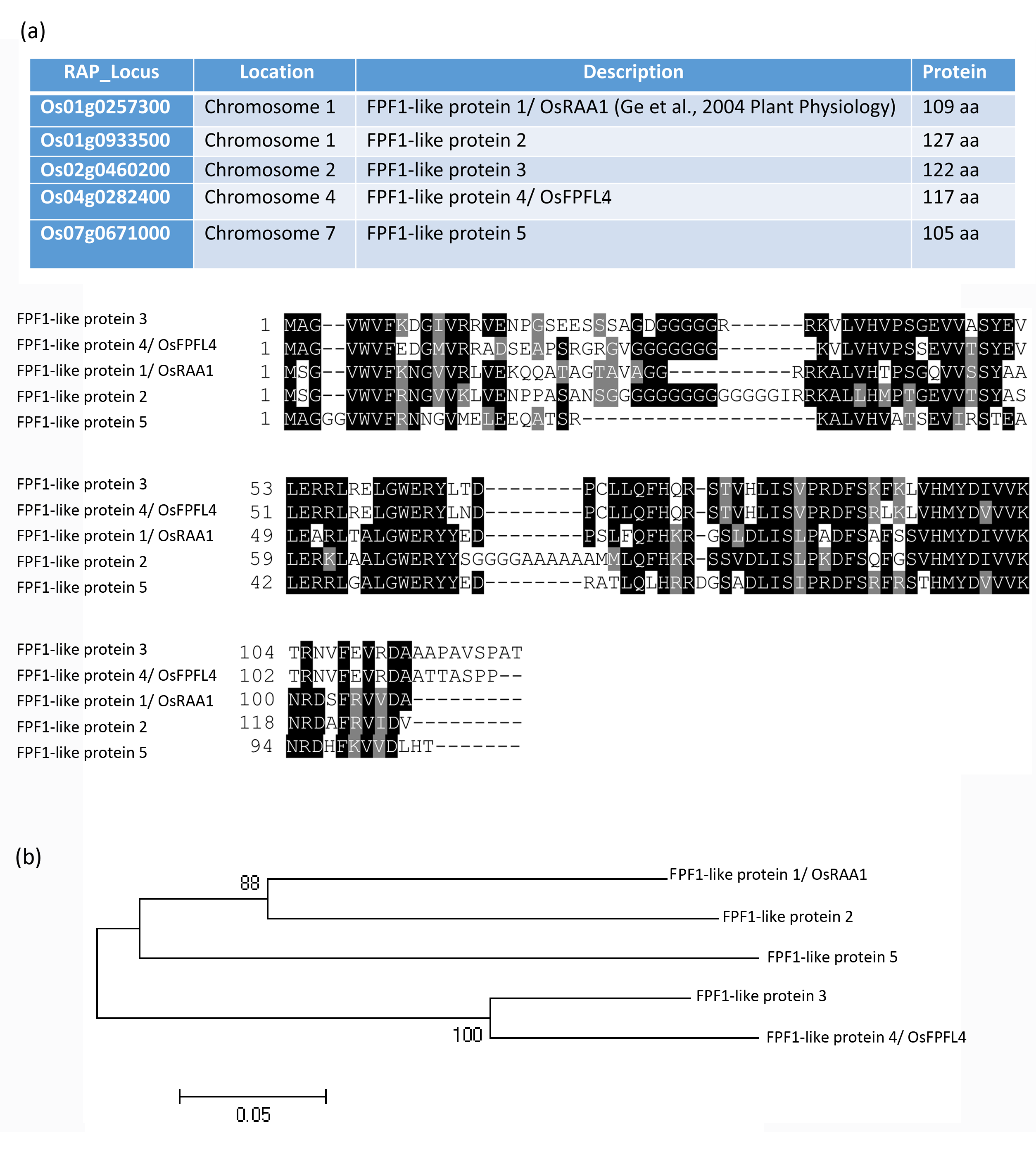

Supplement: Supplementary file 3 — Additional file 3: Figure S1. FPF1-like proteins in rice. [file 12284_2019_364_MOESM3_ESM.tif]
